# PhoPQ two-component regulatory system plays a global regulatory role in antibiotic susceptibility, physiology, stress adaptation, and virulence in *Stenotrophomonas maltophilia*

**DOI:** 10.1186/s12866-020-01989-z

**Published:** 2020-10-14

**Authors:** Hsu-Feng Lu, Bo-Kuan Wu, Yi-Wei Huang, Ming-Zhe Lee, Ming-Fang Li, Hsu-Jung Ho, Hung-Chi Yang, Tsuey-Ching Yang

**Affiliations:** 1 Department of Clinical Pathology, Cheng Hsin General Hospital, Taipei, Taiwan; 2 Department of Restaurant, Hotel and Institutional Management, Fu-Jen Catholic University, New Taipei City, Taiwan; 3 Department of Biotechnology and Laboratory Science in Medicine, National Yang-Ming University, Taipei, Taiwan; 4 Department of Medical Laboratory Science and Biotechnology, Yuanpei University of Medical Technology, Hsinchu, Taiwan

**Keywords:** *Stenotrophomonas maltophilia*, Swimming, Oxidative stress, Antibiotic resistance, Virulence

## Abstract

**Background:**

*Stenotrophomonas maltophilia*, an opportunistic pathogen, is ubiquitously present in various environments, signifying its high capability of environmental adaptation. Two-component regulatory system (TCS) is a powerful implement to help organisms to survive in different environments. In clinic, treatment of *S. maltophilia* infection is difficult because it is naturally resistant to many antibiotics, highlighting the necessity to develop novel drugs or adjuvants. Given their critical and extensively regulatory role, TCS system has been proposed as a convincing target for novel drugs or adjuvants. PhoPQ TCS, a highly conserved TCS in several pathogens, plays crucial roles in low-magnesium adaption, polymyxin resistance, and virulence. In this study, we aimed to characterize the role of PhoPQ TCS of *S. maltophilia* in antibiotic susceptibility, physiology, stress adaptation, and virulence.

**Results:**

To characterize PhoPQ system, *phoP* single mutant as well as *phoP* and *phoQ* double mutant were constructed. Distinct from most *phoPQ* systems of other microorganisms, two features were observed during the construction of *phoP* and *phoQ* single deletion mutant. Firstly, the *phoQ* mutant was not successfully obtained. Secondly, the compromised phenotypes of *phoP* mutant were not reverted by complementing an intact *phoP* gene, but were partially restored by complementing a *phoPQ* operon. Thus, wild-type KJ, *phoP* mutant (KJΔPhoP)*, phoPQ* mutant (KJΔPhoPQ), and complemented strain (KJΔPhoPQ (pPhoPQ)) were used for functional assays, including antibiotic susceptibility, physiology (swimming motility and secreted protease activity), stress adaptation (oxidative, envelope, and iron-depletion stresses), and virulence to *Caenorhabditis elegans*. KJΔPhoPQ totally lost swimming motility, had enhanced secreted protease activity, increased susceptibility to antibiotics (β-lactam, quinolone, aminoglycoside, macrolide, chloramphenicol, and sulfamethoxazole/ trimethoprim), menadione, H_2_O_2_, SDS, and 2,2′-dipyridyl, as well as attenuated virulence to *C. elegans*. Trans-complementation of KJΔPhoPQ with *phoPQ* reverted these altered phenotypes to the wild-type levels.

**Conclusions:**

Given the critical and global roles of PhoPQ TCS in antibiotic susceptibility, physiology, stress adaptation, and virulence, PhoPQ is a potential target for the design of drugs or adjuvants.

## Background

Bacterial survival relies on their ability to cope with the challenge of environmental pressures. The two-component regulatory system (TCS) is a multi-variable regulatory mechanism, which is widely present in bacteria and contributes to the adaptation of bacteria to environmental stress. In a typical prokaryotic TCS, an environmental stimulus is sensed by the sensor kinase (SK), leading to its autophosphorylation, and transfer of the phosphoryl group of SK to the cognate response regulator (RR). The activated RR binds to the promoters of the target genes, ultimately leading to coordinated changes in global gene expression profiles [1, 2].

The PhoP/PhoQ (PhoPQ) TCS system is highly conserved among many pathogenic or non-pathogenic gram-negative bacteria [3]. The PhoPQ TCS consists of the SK PhoQ and the RR PhoP. PhoQ can respond to several environmental signals by autophosphorylation, including low-Mg^2+^ and low-Ca^2+^ conditions, acidic pH, the presence of cationic antimicrobial peptides (CAP), and osmotic upshift [4–7]. Phosphorylated PhoQ transfers the phosphate to PhoP, and activated PhoP further regulates the expression of downstream genes (PhoP regulon). Although PhoQs in different microorganisms can sense the same or similar environmental signals, the regulons triggered by phosphorylated PhoP vary in different species of bacteria [8, 9]. The PhoPQ systems of human pathogens have been shown to participate in low-magnesium adaptation, acid tolerance, polymyxin resistance, and virulence [10–12]. Given the extensive coverage of PhoP regulon, loss of function of PhoPQ generally has a pleiotropic impact on a bacterium [3].

*Stenotrophomonas maltophilia* is a gram-negative, ubiquitous environmental bacterium, which can be isolated from water, soils, plant roots, animals, and humans. It is equipped with a wide range of activities, including plant growth promotion, pollutants breakdown, secondary metabolites production, and human pathogenicity [13]. *S. maltophilia* is not a highly virulent pathogen, but it has emerged as an important opportunistic pathogen. Chronic infections by this organism are prevalent in immunocompromised or cystic fibrosis patients. *S. maltophilia* infections are difficult to treat, as it is intrinsically resistant to various antibiotics [14].

Given the notable diversity of its habitats, *S. maltophilia* harbors an array of systems to adapt to different environmental niches. Sequencing of the *S. maltophilia* genome revealed the presence of at least 43 TCS regulatory proteins [15], implying that the ability of *S. maltophilia* to adapt to different ecological niches is noteworthy and the underlying mechanisms are very complex. Despite the fact that the significance of PhoPQ TCS in other bacteria have been widely reported [3], the functions of PhoPQ TCS in *S. maltophilia* have not been thoroughly examined except a recent report by Liu’s group [12]. Liu’s group has demonstrated that PhoPQ TCS of *S. maltophilia* is involved in the resistance to aminoglycoside and polymyxin, as well as the membrane permeability [12]. Therefore, in this study we examined the role of PhoPQ TCS in antibiotic susceptibility, physiological functions, stress adaptation, and virulence to nematode.

## Results

### Role of phoPQ TCS in bacterial viability in magnesium-limited condition

To study the functions of *phoPQ* in *S. maltophilia*, in frame deletions were created at the *phoP* and *phoQ* loci, either individually or in combination, by double cross-over homologous recombination, yielding KJΔPhoP and KJΔPhoPQ. Despite several attempts, a *phoQ* mutant was not constructed.

**Fig. 1:**
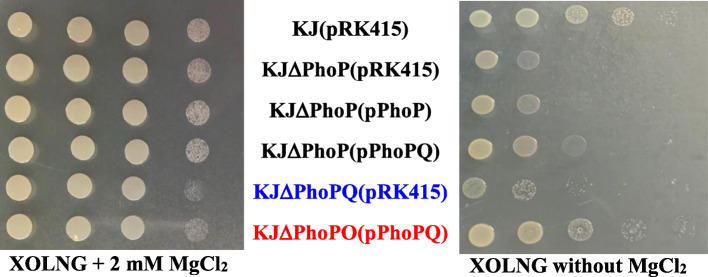
Impact of *phoPQ* TCS on bacterial growth in magnesium limited condition. The logarithmic-phase bacterial cells of 2 × 10^5^ CFU/μl were 10-fold serially diluted. Bacterial suspension (5 μl) was spotted onto the minimal medium XOLNG with or without MgCl_2_. After 24 h of incubation at 37 °C, the growth of bacterial cells was observed

In *S. maltophilia* and in numerous other bacteria, the PhoPQ TCS is activated by limiting the availability of magnesium and the *phoP* mutant shows growth impairment in this condition [10, 12]. We confirmed impairment of the growth of KJΔPhoP and KJΔPhoPQ on minimal XOLNG medium without MgCl_2_. The complementation of KJΔPhoP with plasmid pPhoPQ partially restored the growth defect of KJΔPhoP but pPhoPQ complementation in KJΔPhoPQ fully restored its growth. However, complementation of KJΔPhoP with plasmid pPhoP did not restore the growth defect (Fig. 1).

**Fig. 2:**
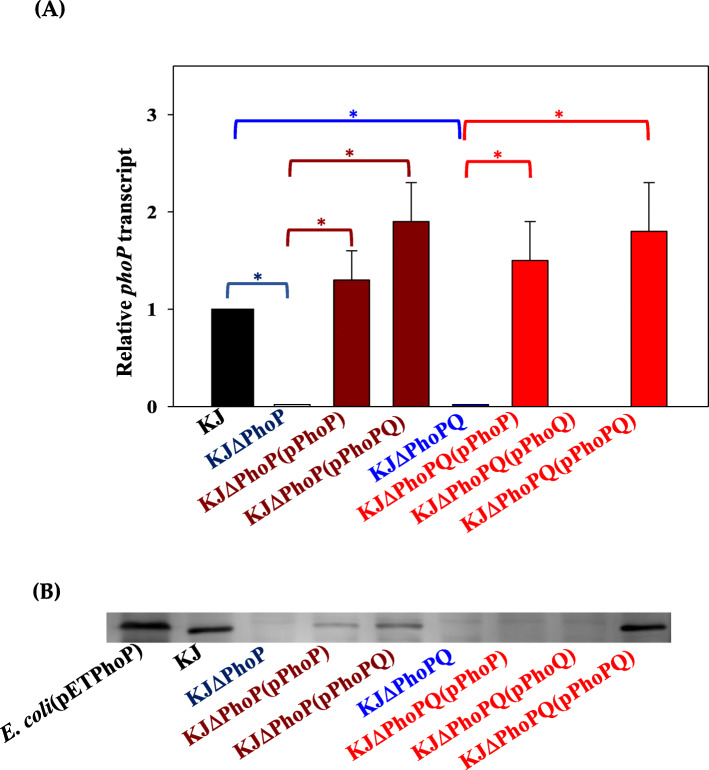
Expression of *PhoP* transcript and PhoP protein in wild-type KJ, its derived mutants, and the complementary strains. **a** *PhoP* transcript levels. Overnight culture of bacterial cells was inoculated into fresh LB with an initial OD_450nm_ of 0.15. Cells were grown aerobically for 5 h before measuring *phoP* transcript using qRT-PCR. Bars represent the average values from three independent experiments. Error bars represent the standard error of the mean. *, *P* < 0.01, significance calculated by Student’s *t* test. **b** PhoP protein levels. Whole bacterial cell lysates were denatured and separated by 14% SDS-PAGE and then transferred onto polyvinylidene difluoride (PVDF) membranes. The membrane was incubated with anti-PhoP antibodies and secondary mouse anti-rabbit IgG immunoglobulin conjugated to horseradish peroxidase. The reaction was developed with a mixture of diamino benzidine and 30% hydrogen peroxide. Full-length blots/gels are presented in Supplementary’s Figure S1

To explain the failure of *phoP* complementation assay in KJΔPhoP, *phoP* expression in the KJΔPhoP (pPhoP) was evaluated. While the *phoP* transcript was expressed in KJΔPhoP (pPhoP) and KJΔPhoP (pPhoPQ) (Fig. 2a), only marginal to moderate levels of PhoP protein were detected in both strains (Fig. 2b; Fig. S1). Similar assessment was also performed in KJΔPhoPQ by complementation of plasmids pPhoP, pPhoQ, and pPhoPQ, respectively. The same trend was observed that abundant *phoP* transcript, but not PhoP protein, was detected in KJΔPhoPQ (pPhoP); however, both *phoP* transcript and PhoP protein reached significant levels in KJΔPhoPQ (pPhoPQ) (Fig. 2a & b). This provided a reasonable explanation for the failure of *phoP* complementation in KJΔPhoP. It was worthily mentioned that there is a slight difference of migration of the PhoP proteins in the different lanes shown in Fig. 2b. This might be related to the phosphorylation status of PhoP proteins. The underlying mechanism for the absence of PhoP proteins in KJΔPhoP (pPhoP) and KJΔPhoPQ (pPhoP) is not immediately clear at present. Thus, the *phoP* complementation strains were not included in the following study.

### Role of PhoPQ TCS in antibiotic susceptibility

The susceptibility of *phoP* or *phoPQ* mutants to antibiotics ceftazidime, ticarcillin/clavulanic acid, ciprofloxacin, levofloxacin, nalidixic acid, kanamycin, tobramycin, erythromycin, leucomycin, chloramphenicol, and trimethoprim-sulfamethoxazole (SXT) was examined. *PhoP* or *PhoPQ* deletion reduced the MICs by 4- to 64-fold in all the antibiotics tested. Trans-complementation of KJΔPhoPQ with pPhoPQ restored resistance to all tested antibiotics (Table 1).

**Table 1:** Antibiotic susceptibilities of *S. maltophilia* KJ, KJΔPhoPQ, and KJΔPhoPQ (pPhoPQ)

Antibiotic	MIC (μg/ml)			
	KJ (pRK415)	KJΔPhoP (pRK415)	KJΔPhoPQ (pRK415)	KJΔPhoPQ (pPhoPQ)
**β-lactam**				
Ceftazidime	256	64	16	256
Ticarcillin-clavulanate	128	16	8	128
**Quinolone**				
Ciprofloxacin	1	0.25	0.125	1
Levofloxacin	1	0.125	0.125	1
Nalidixic acid	8	2	2	8
**Aminoglycoside**				
Kanamycin	256	4	4	128
Tobramycin	256	4	4	128
**Macrolide**				
Erythromycin	64	16	8	64
Leucomycin	256	32	32	256
Chloramphenicol	8	2	2	8
Sulfamethoxazole/trimethoprim	2	0.5	0.25	2

### Role of PhoPQ TCS in bacterial physiology

**Fig. 3:**
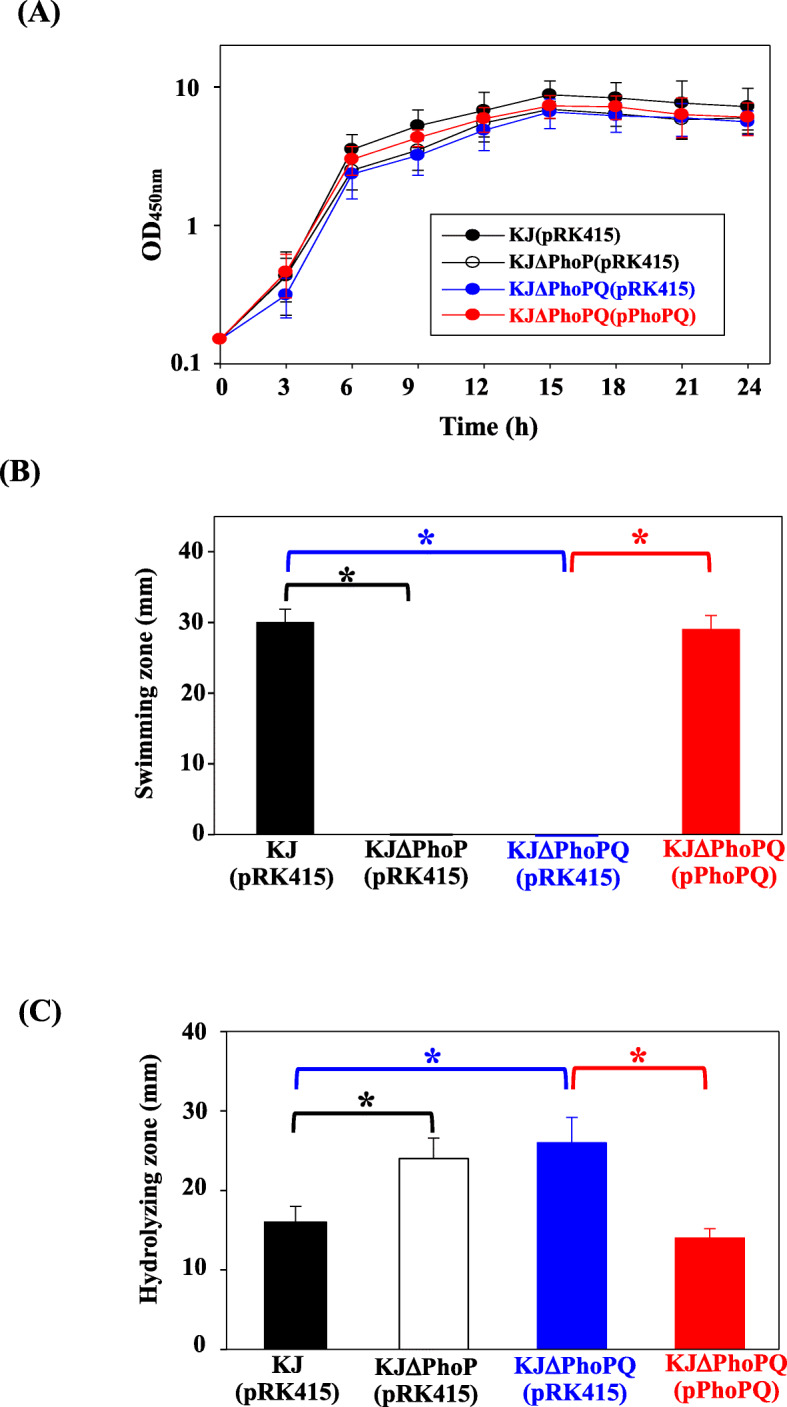
Roles of phoPQ TCS in physiological functions. **a** Roles of *phoPQ* TCS in bacterial growth. Overnight culture of bacterial cells was inoculated into fresh LB broth with an initial OD_450nm_ of 0.15. The bacterial growth was monitored by recording the OD_450nm_ every 3 h. Error bars represent the standard error of the mean. *, *P* < 0.01, significance calculated by Student’s *t* test. **b** Roles of *phoPQ* TCS in swimming motility. The logarithmic-phase bacterial culture was adjusted to OD_450nm_ of 1.0. Five-microliter suspension was inoculated into the swimming agar (1% tryptone, 0.5% NaCl, and 0.15% agar) and the swimming zones were recorded after 48-h incubation at 37 °C. Bars represent the average values from three independent experiments. Error bars represent the standard error of the mean. *, *P* < 0.01, significance calculated by Student’s *t* test. **c** Roles of *phoPQ* TCS in secreted protease activity. The logarithmic-phase bacterial culture was adjusted to OD_450nm_ of 1.0 and 40 μl bacterial aliquot were inoculated into the skim milk agar plate. The proteolytic activity of bacteria was assessed by measuring the transparent zones around the bacteria after incubation for 72 h at 37 °C. Bars represent the average values from three independent experiments. Error bars represent the standard error of the mean. *, *P* < 0.01, significance calculated by Student’s *t* test

The effects of the inactivation of *phoP* or *phoPQ* on bacterial physiology were assessed with respect to bacterial growth, swimming, and secreted protease activity. Bacterial growth was evaluated by monitoring the OD_450nm_ at different growth time points. KJΔPhoP and KJΔPhoPQ had reduced, but not statistically significant, growth compared to wild-type KJ (Fig. 3a).

Next, the swimming motilities of KJΔPhoP and KJΔPhoPQ were evaluated. Inactivation of *phoP* or *phoPQ* of *S. maltophilia* KJ completely abolished the swimming motility. Complementation of KJΔPQ with pPhoPQ restored swimming motility to the wild-type level (Fig. 3b & Fig. S2).

The secreted protease activities of KJ, KJΔPhoP, and KJΔPhoPQ were determined. Inactivation of *phoP* or *phoPQ* of strain KJ resulted in higher secreted protease activity and pPhoPQ complementation restored the secreted protease activity to the wild-type level (Fig. 3c & Fig. S3).

### Role of PhoPQ TCS in stress adaptation

The contribution of *phoP* or *phoPQ* toward adaptation to oxidative, envelope, and iron-depleted stresses was investigated.

**Fig. 4:**
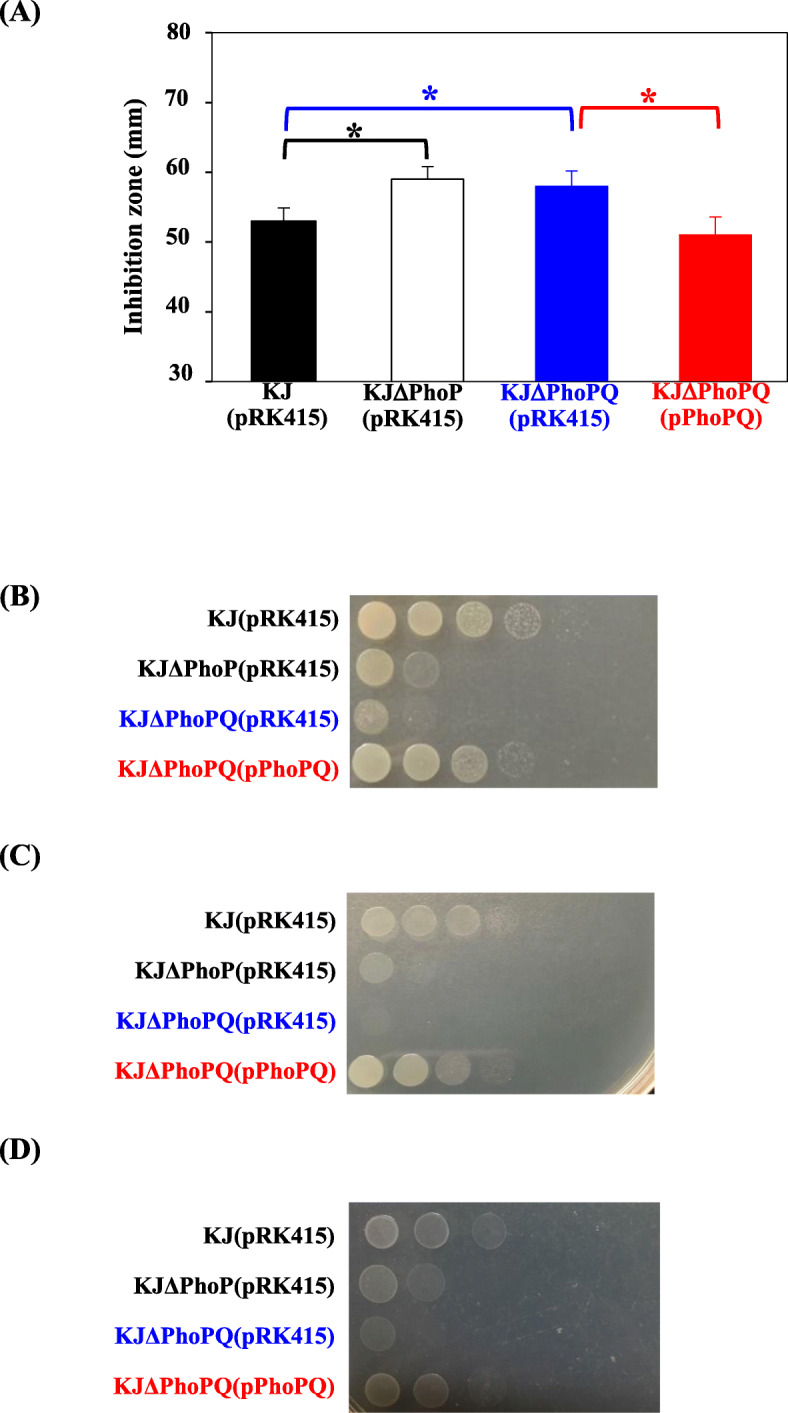
Roles of *phoPQ* TCS in stress adaptation. **a** Roles of *phoPQ* TCS in H_2_O_2_ susceptibility. LB agar was uniformly spread with the bacterial cell suspension tested. Sterile filter paper soaked in 15 μl of 20% H_2_O_2_ was placed onto the center of agar. The diameter of growth inhibition zone was measured after 24-h incubation at 37 °C. Bars represent the average values from three independent experiments. Error bars represent the standard error of the mean. *, *P* < 0.01, significance calculated by Student’s *t* test. **b** Roles of *phoPQ* TCS in MD susceptibility. The logarithmic-phase bacterial culture was adjusted to an OD_450nm_ of 1.0 and serially diluted 10-fold. Five-microliter suspensions were applied onto the LB agar containing 50 μg/ml MD. After 24-h incubation at 37 °C, cell viability was imaged. **c** Roles of *phoPQ* TCS in SDS susceptibility. The logarithmic-phase bacterial culture was adjusted to an OD_450nm_ of 1.0 and serially diluted 10-fold. Five-microliter suspensions were applied onto the LB agar containing 0.01% SDS. After 24-h incubation at 37 °C, cell viability was imaged. **d** Roles of *phoPQ* TCS in iron depletion tolerance. The logarithmic-phase bacterial culture was adjusted to OD_450nm_ of 1.0 and serially diluted 10-fold. Five-microliter suspensions were applied onto the LB agar containing 45 μg/ml DIP. After 24-h incubation at 37 °C, cell viability was imaged

The role of *phoP* or *phoPQ* in oxidative stress response was tested by H_2_O_2_ and menadione (MD) susceptibility assays. KJΔPhoP and KJΔPhoPQ were more susceptible to H_2_O_2_ and MD than wild-type KJ. The H_2_O_2_ and MD tolerances of KJΔPhoPQ were reverted by complementing with the pPhoPQ plasmid (Fig. 4a & b).

The tolerances to envelope stress and iron depletion stress were assessed by examining cell viability in sodium dodecyl sulfate (SDS)-containing and 2,2′-dipyridyl (DIP)-containing LB agars, respectively. The viabilities of KJΔPhoP and KJΔPhoPQ were apparently compromised compared to that of wild-KJ. Complementation of KJΔPhoPQ with pPhoPQ restored cell viability (Fig. 4c & d).

### Role of PhoPQ TCS in virulence to nematode

**Fig. 5:**
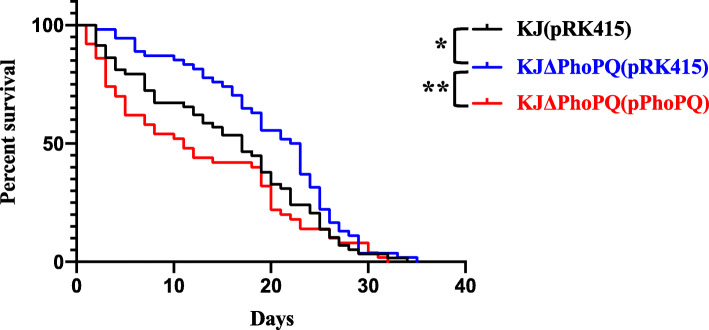
Role of *phoPQ* in *C. elegans* survival. Sixty worms at the L4 stage were transferred to *S. maltophilia*-seeded NGM agar plates and scored for survival daily

The relationship between PhoPQ TCS and virulence was assessed using the *Caenorhabditis elegans* model. Median survivals for KJ (pRK415), KJΔPhoPQ (pRK415), and KJΔPhoPQ (PhoPQ) were 17, 22.5, and 11 days, respectively (Fig. 5). KJΔPhoPQ was less virulent to *C. elegans* than wild-type KJ, and trans-complementation of KJΔPhoPQ with plasmid pPhoPQ restored virulence to *C. elegans*.

### PhoPQ TCS regulates the MD-mediated upexpression of *sodA1*, *katA2*, and *katE*

**Fig. 6:**
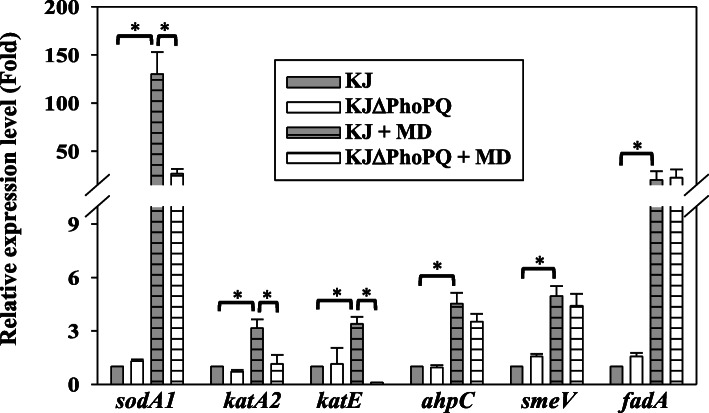
The regulatory role of PhoPQ TCS in the expression of *sodA1*, *katA2*, *katE*, *ahpC*, *smeV*, and *fadA* genes. Overnight culture of KJ and KJΔPhoPQ cells was inoculated into fresh LB broth without or with MD at an initial OD_450nm_ of 0.15. Cells were grown aerobically for 5 h before measuring *sodA1*, *katA2*, *katE*, *ahpC*, *smeV*, and *fadA* transcripts using qRT-PCR. All values were normalized to the transcript of MD-non-treated KJ cells. Bars represent the average values from three independent experiments. Error bars represent the standard error of the mean. *, *P* < 0.001, significance calculated by Student’s t test

We were interested in elucidating the possible PhoPQ-regulated candidate genes involved in the above phenotypes. Of the phenotypes assayed, oxidative stress alleviation was selected for following study since the underlying mechanisms involved in MD-mediated oxidative stress alleviation of *S. maltophilia* are extensively surveyed in our previous studies [16–21]. The known mechanisms against oxidative stress in *S. maltophilia* include superoxide dismutases (SodA1, SodA2, SodB, and SodC1C2) [16], catalases (KatA1, KatA2, KatE, and KatMn), alkyl hydroperoxide reductase (AhpC) [17], efflux pumps (MacABCsm, SmeYZ, and SmeVWX) [18–20], and the formaldehyde detoxification system FadACB [21]. KJ and KJΔPhoPQ were cultured in the LB broth without or with MD, and the expressions of these oxidative stress-associated genes were assessed by qRT-PCR. Of thirteen genes surveyed, six genes (*sodA1*, *katA2*, *katE*, *ahpC*, *smeV*, and *fadA)* in wild-type KJ were upregulated upon MD challenge (Fig. S4). The expressions of the six upregulated genes in KJΔPhoPQ with or without MD treatment were further assessed. In the absence of MD, the six transcripts in KJ and KJΔPhoPQ were comparable. Compared to those in MD-treated KJ cells, the *sodA1, katA2,* and *katE* transcript levels were apparently dropped in MD-treated KJΔPhoPQ cells (Fig. 6).

## Discussion

PhoPQ is a highly conserved TCS in several gram-negative microbes. Compared to several known PhoPQ TCSs of other gram-negative human pathogens, the PhoPQ TCS of *S. maltophilia* has some special features, as revealed in this study. (i) The *phoQ* mutant cannot be obtained. (ii) The complementary plasmid pPhoP can be successfully transcribed in KJΔPhoP, but PhoP protein is hardly detected in significant levels (Fig. 2). (iii) Compared to known PhoPQ systems, the functions of PhoPQ system of *S. maltophilia* are the most comprehensive, contributing to antibiotic susceptibility, physiology, stress adaptation, and virulence.

The unavailability of the *phoQ* mutant in this study was reminiscent of the essentiality of PhoPQ in *Xanthomonas campestris* [22]. With the exemption of *Xanthomonas campestris*, the reported *phoQ* orthologs in other bacteria, such as *Escherichia coli*, *Salmonella enterica*, *Pseudomonas aeruginosa*, and *P. fluorescens*, are mutable [8, 23, 24]. Peng et al. have proposed the polygenic transcriptional rewiring model to explain the essentiality of PhoPQ in *X. campestris*. They suggested that the PhoP regulon of *X. campestris* includes several essential genes, but the PhoP regulons of other bacteria do not [22]. In contrast to PhoP essentiality in *X. campestris*, the *phoP* deletion mutant is viable in *S. maltophilia*. Thus, we proposed two possibilities for explanation. (i) PhoQ may interact with other response regulator(s) to globally activate several regulatory circuits, in addition to its original cognate PhoP. In several gram-negative bacteria, it is common to have cross-talk between noncognate pairs of sensor kinase and response regulator. The PhoQ-associated regulons may include several genes essential for the viability of *S. maltophilia*. (ii) Non-phosporylated PhoP may have a toxic effect and inhibit growth.

A peculiar observation, which has not been reported in other species, is that, in contrast to pPhoPQ, the plasmid pPhoP cannot successfully complement the compromised phenotype of KJΔPhoP. Similar experiment design has been observed by Liu et al., who complemented a *phoP* mutant of *S. maltophilia* S22 with a *phoPQ*-containing plasmid, but not with a *phoP*-containing plasmid [12]. They demonstrated that *phoP* inactivation causes a polar effect on the expression of downstream *phoQ* gene [12]. In the present study, we verified that *phoP* transcript, but not sufficient PhoP protein, was detectable in KJΔPhoP (pPhoP) and KJΔPhoPQ (pPhoP), while both the *phoP* transcript and PhoP protein were detectable in KJΔPhoPQ (pPhoPQ) (Fig. 2). It seemed that an intact *phoP-phoQ* transcript is critical for a successful translation of the *phoPQ* transcript. Inactivation of *phoP* has a negative impact on the stability of *phoQ* transcript [12]. On the other hand, loss-of-function of *phoQ* significantly decreased the PhoP protein levels (Fig. 2b), implying the involvement of PhoQ in stability of PhoP protein. The complex interplay between the stability of *phoP-phoQ* transcript and the functions of PhoP and PhoQ proteins is needed to be further elucidated.

The contribution of the PhoPQ system to antibiotic resistance has been reported in other gram-negative bacteria, but was limited to polymyxin and tetracycline [1, 23, 25]. A notable observation of this study is that the PhoPQ of *S. maltophilia* had a marked impact on resistance to all the antibiotics tested (Table 1). Of all reported PhoPQ systems in gram-negative bacteria, the contribution of the *S. maltophilia* PhoPQ TCS to antibiotic resistance is the most comprehensive and prominent.

Enzymatic hydrolysis and efflux pump extrusion are two known oxidative stress alleviation systems for Gram-negative bacteria to deal with oxidative stress [26, 27]. In addition, a formaldehyde detoxification system, FadACB, has recently been reported to contribute to oxidative stress alleviation in *S. maltophilia* [21]. SoxR and OxyR are two well-known regulators involved in the regulatory circuits [28]. In this article, we further extended the understanding in the regulation of oxidative stress alleviation systems in *S. maltophilia*. PhoPQ TCS participates in the MD-mediated oxidative stress alleviation by upregulating the expression of *sodA1*, *katA2*, and *katE* (Fig. 6). Since the consensus sequence for PhoP binding sites of *S. maltophilia* is not clear right now, we tried to survey whether the promoter regions of the three genes have conserved PhoP binding sites referenced from the reported PhoP boxes of *E. coli* and *P. aeruginosa* [29, 30] and on positive results were obtained.

For a successful colonization or infection, a bacterium should be able to invade host cells and have the functional capacity to defend itself from host-imposed stresses. Swimming motility is a critical ability for bacterial accessibility to host cells and the secreted protease activity is required to damage the host epithelial barrier and resist the action of host’s virulence factors [31]. Innate/adaptive immunity and nutritional immunity are host defense mechanisms against pathogen invasion. ROS-mediated oxidative stress, cationic antimicrobial peptides-mediated envelope stress, magnesium depletion and iron depletion are common stresses imposed by host cells. The PhoPQ system of *S. maltophilia* governs swimming and adaption to oxidative stress, envelope stress, and iron depletion; thus, a functional PhoPQ system is critical for a successful infection.

## Conclusions

The PhoPQ system of *S. maltophilia* has been demonstrated involvement in the resistance to polymyxin B, chloramphenicol, aminoglycoside, and ampicillin [12]. In this study, we further extended the understanding of PhoPQ function in the resistance to ceftazidime, ticarcillin-clavulanate, quinolone, and sulfamethoxazole/trimethoprim, which are effective antibiotics commonly used for the treatment of *S. maltophilia* infection [14]. Furthermore, PhoPQ system also contributes to swimming, stress adaptation (oxidative stress, envelope stress, and iron depletion), and virulence to *C. elegans*. These findings signify the potential of PhoPQ as a target for drug design or adjuvant therapy.

## Methods

### Bacterial strains, media, plasmids, and primers

Table S1 lists the bacterial strains, plasmids, and primers used in this study. The XOLNG minimal medium was prepared as described previously [32].

### Construction of in-frame deletion mutants

The DNA fragments flanking the deleted genes were amplified by PCR and subsequently cloned into pEX18Tc to generate the mutagenic plasmids for double cross-over homologous recombination. The primers sets for the construction of mutagenic plasmids were PhoPN-F/PhoPN-R and PhoQN-F/PhoQN-R for pΔPhoP, PhoQN-F/PhoQN-R and PhoQC-F/PhoQC-R for pΔPhoQ, and PhoPN-F/PhoPN-R and PhoQC-F/PhoQC-R for pΔPhoPQ (Table S1). These of mutagenic plasmids were transferred into *S. maltophilia* by conjugation. The procedures for conjugation, transconjugants selection, and confirmation of mutants correctness were carried out as described previously [33].

### Construction of *phoP*, *phoQ*, and *phoPQ* complementary plasmids, pPhoP, pPhoQ, and pPhoPQ

To obtain expression plasmids for complementation experiments, *phoP, phoQ,* and *phoP-phoQ* genes were amplified by PCR from strain KJ and cloned into pRK415. The used primer sets were PhoP-F/PhoP-R for *phoP* gene, PhoQ-F/PhoQ-R for *phoQ* gene, and PhoPQ-F/PhoPQ-R for *phoP-phoQ* genes (Table S1).

### Quantitative reverse transcription-PCR (qRT-PCR)

The DNA-free RNA of bacterial cells was extracted using Total RNA Extraction Kit Mini (ARROWTEC) and reverse transcribed to into cDNA as described previously [17]. qRT-PCR was carried out by ABI Prism 7000 Sequence Detection System (Applied Biosystems, Foster City, CA, United States). The primers used for qRT-PCR are listed in Table S1. The relative levels of expression were calculated by the threshold cycle *ΔΔCt* method [34] using the 16S rRNA as the control. Each assay was independently performed at least three times.

### Preparation of polyclonal anti-rabbit PhoP antibody

The *phoP* gene of *S. maltophilia* KJ was obtained by PCR using the primers PhoPHis-F and PhoPHis-R (Table S1) and then inserted into the *E. coli* expression vector pET30b, yielding pETPhoP. The *E. coli* BL21(DE3) was transformed with plasmid pETPhoP. Overnight-cultured *E. coli* BL21(pETPhoP) was subcultured into fresh LB broth and grown to logarithmic growth phase, IPTG was added at final concentrations of 1 mM for 3 h. Bacterial cells were disrupted by sonicating and cell debris were removed by centrifugation. The Recombinant PhoP-6xHis proteins were obtained from clear lysate with Ni-NTA resin. Purity of PhoP-6xHis was determined by SDS-PAGE. Antibodies against PhoP-6xHis protein were raised by immunization of New Zealand rabbits with the purified PhoP-6xHis protein.

### Western blotting analysis

Whole bacterial cell lysates were denatured and separated by 14% SDS-PAGE and then transferred onto polyvinylidene difluoride (PVDF) membranes. The blot was reacted with anti-PhoP antibodies and secondary mouse anti-rabbit IgG immunoglobulin conjugated to horseradish peroxidase (Sigma Chemical Co., St. Louis, MO).

### Antibiotics susceptibility test

Antimicrobial susceptibility test was performed using the twofold agar dilution method on Mueller-Hinton agar according to Clinical and Laboratory Standards Institute (CLSI) guidelines [35]. Bacterial growth was recorded after incubation at 37 °C for 16–18 h. The minimal inhibitory concentration (MIC) was the lowest concentration of the antibiotic that completely inhibited bacterial growth. Antibiotics were purchased from Sigma Chemical Co. Each assay was independently performed at least three times.

### Swimming motility

Overnight culture was adjusted to OD_450_ of 1.0 and 5 μL aliquot was inoculated onto the surface of swimming agar (1% tryptone, 0.5% NaCl, and 0.15% agar). Plates were incubated at 37 °C for 48 h and the diameter (mm) of the migration zone of bacteria was measured [19]. Each assay was independently performed at least three times.

### Secreted protease activity assay

The 1% skin milk-supplemented LB agar was prepared for secreted protease activity assay, with a 6-mm-diameter hole in the center for the convenience of bacterial loading. Bacterial culture tested was adjusted to an OD450 of 1.0 and 40 μL aliquot was dripped onto the hole of the skim milk agar plates. The proteolytic activity of bacteria was assessed by measuring the transparent zones around the bacteria after 72 h-incubation at 37 °C [19]. Each assay was independently performed at least three times.

### Menadione (MD), sodium dodecyl sulfate (SDS), and 2,2′-dipyridyl (DIP) susceptibility assay

The logarithmic-phase bacterial culture was adjusted to an OD450nm of 1 and subsequently 10-fold serially diluted. Five microliter aliquot was spotted onto the LB agars containing 50 mg/L MD, 0.01% SDS, and 45 mg/L DIP, respectively. Cell viabilities were recorded after 24-h incubation at 37 °C. Each assay was independently performed at least three times and the pictures in the results was a representative.

### H_2_O_2_ sensitivity assay

The logarithmic-phase bacterial cells of OD_450nm_ 1.0 were spread evenly on LB agar plates. Filter paper disks (6 mm) impregnated with 15 μl 20% H_2_O_2_ were placed onto the top of agar plate. After 24-h incubation at 37 °C, the growth inhibition zone was measured [19]. Each assay was independently performed at least three times.

### *Caenorhabditis* elegans survival assay

N2 (wild type) *C. elegans* was acquired from Caenorhabditis Genetics Center (University of Minnesota, Minneapolis, USA). *C. elegans* was maintained at 20 °C based on standard protocols [36]. The assay was carried out with 60 worms at the L4 stage and scored for survival over time. In brief, synchronized L1 larvae were obtained from gravid adults by bleach based on a previous protocol [37]. The L1 larvae were cultured on *E. coli HT115* until L4 stage before transferring to *S. maltophilia*-seeded NGM agar plates supplemented with 50 μM fluorodeoxyuridine (FUdR) to maintain a synchronous worm population [38]. *S. maltophilia* strains were grown overnight in LB and spread (3 × 10^5^ CFU/cm^2^) onto NGM agar plates followed by overnight incubation at 37 °C. The worms exposed to *S. maltophilia* were scored for survival daily. During daily transfer of worms, fresh NGM agar plates seeded with sufficient bacterial food was provided regularly, which excluded the possibility that the survival of worms might be affected by the lack of food. *C. elegans* survival assay was analyzed by using GraphPad Prism version 8.3 (GraphPad Software, San Diego). Kaplan-Meier log rank analysis was used to determine the difference between *C. elegans* exposed to *S. maltophilia* strains. Each assay was independently performed at least three times.

## Supplementary information

**Additional file 1: Table S1.** Bacterial strains, plasmids, and primers used in this study.

**Additional file 2: Figure. S1.** SDS-PAGE and Western blotting of total bacterial proteins of *S. maltophilia* KJ, its derived mutants, and complementary strains. Overnight culture of bacterial cells was inoculated into fresh LB with an initial OD_450nm_ of 0.15. Cells were grown aerobically for 5 h. Whole bacterial cell lysates were denatured and separated by 14% SDS-PAGE. (A) The gel was stained with 0.1% Coomassie Blue R250 in 10% acetic acid, 50% methanol, and 40% H_2_O for 30 min, and then destained with 10% acetic acid, 50% methanol, and 40% H_2_O until the background was clear. (B) The proteins in the gel were transferred onto polyvinylidene difluoride (PVDF) membranes. The blot was reacted with anti-PhoP antibodies and secondary mouse anti-rabbit IgG immunoglobulin conjugated to horseradish peroxidase (Sigma Chemical Co., St. Louis, MO). M, protein markers; lane 1, *E. coli* (pETPhoP); lane 2, KJ; lane 3, KJΔPhoP; lane 4, KJΔPhoP (pPhoP); lane 5, KJΔPhoP (pPhoPQ); lane 6, KJΔPhoPQ; lane 7, KJΔPhoPQ (pPhoP); lane 8, KJΔPhoPQ (pPhoQ); lane 9, KJΔPhoPQ (pPhoPQ). The cropped image presented in Fig. 2b is marked by a black rectangle.

**Additional file 3: Figure S2.** The swimming motility of wild-type KJ, *phoP* mutant (KJΔPhoP), *phoPQ* mutant (KJΔPhoPQ), and complementation strain (KJΔPhoPQ (pPhoPQ)). The logarithmic-phase bacterial culture was adjusted to OD_450nm_ of 1.0. Five-microliter suspension was inoculated into the swimming agar (1% tryptone, 0.5% NaCl, and 0.15% agar) and the swimming zones were recorded after 48-h incubation at 37 °C.

**Additional file 4: Figure S3.** The secreted protease activity of wild-type KJ, *phoP* mutant (KJΔPhoP), *phoPQ* mutant (KJΔPhoPQ), and complementation strain (KJΔPhoPQ (pPhoPQ)). The logarithmic-phase of bacterial culture was adjusted to OD_450nm_ of 1.0 and 40 μl bacterial aliquot were inoculated into the skim milk agar plate. The proteolytic activity of bacteria was assessed by measuring the transparent zones around the bacteria after incubation for 72 h at 37 °C.

**Additional file 5: Figure S4.** The expression of oxidative stress alleviation-associated genes of *S. maltophilia* KJ in response to MD challenge. Overnight culture of KJ cells was inoculated into fresh LB broth without or with MD (16 μg/ml) at an initial OD_450nm_ of 0.15. Cells were grown aerobically for 5 h before measuring the indicated transcripts using qRT-PCR. All values were normalized to the transcript of MD-non-treated KJ cells. Bars represent the average values from three independent experiments. Error bars represent the standard error of the mean. *, *P* < 0.001, significance calculated by Student’s t test.

## Data Availability

The datasets used and/or analysed during the current study available from the corresponding author on reasonable request.

## Abbreviations

TCS: Two-component regulatory system
SK: Sensor kinase
RR: Response regulator
CAP: Cationic antimicrobial peptides
SDS: Sodium dodecyl sulfate
DIP: 2,2′-dipyridyl
MD: Menadione
qRT-PCR: Quantitative reverse transcription-PCR
