# Supplementary material for: PhoPQ two-component regulatory system plays a global regulatory role in antibiotic susceptibility, physiology, stress adaptation, and virulence in Stenotrophomonas maltophilia
Source: BMC Microbiol. 2020 Oct 14;20:312. doi: 10.1186/s12866-020-01989-z (PMC7559202; doi:10.1186/s12866-020-01989-z)
Supplement: Supplementary file 1 — Additional file 1: Table S1. Bacterial strains, plasmids, and primers used in this study. [file 12866_2020_1989_MOESM1_ESM.docx]

**Table S1. Bacterial strains, plasmids, and primers used in this study**

| Strain, plasmid, or primer | Genotype or properties | Reference |
| --- | --- | --- |
| ***S. maltophilia***  KJ | A clinical *S. maltophilia* isolate | Hu et al., 2008 |
| KJΔPhoP | *S. maltophilia* KJ mutant of *phoP* gene; *ΔphoP* | This study |
| KJΔPhoPQ | *S. maltophilia* KJ mutant of *phoPQ* genes; *ΔphoPQ* | This study |
| ***E. coli*** |  |  |
| DH5α | F- φ80d/*acZΔM15* Δ(*lacZYA-argF*)*U169* *deoR recA1 endA1 hsdR17* (r_k_^-^ m_k_^+^) *phoA supE44λ* *thi-1 gyrA96 relA1* | Invitrogen |
| S17-1 | λ*pir*^+^ mating strain | Simon et al., 1986 |
| **Plasmids** |  |  |
| pEX18Tc | *sacB oriT*, Tc^r^ | Hoang et al., 1998 |
| pRK415 | Mobilizable broad-host-range plasmid cloning vector, RK2 origin; Tc^r^ | Keen et al., 1998 |
| pΔPhoP | pEX18Tc with an internal-deletion *phoP* gene; Tc^r^ | This study |
| pΔPhoPQ | pEX18Tc with partial N-terminus of *phoP* gene and partial C-terminus of *phoQ* gene; Tc^r^ | This study |
| pPhoP  pPhoQ | pRK415 with an intact *phoP* gene of strain KJ; Tc^r^  pRK415 with an intact *phoQ* gene of strain KJ; Tc^r^ | This study  This study |
| pPhoPQ | pRK415 with intact *phoP* and *PhoQ* genes of strain KJ; Tc^r^ | This study |
| **Primers**  PhoPHis-F  PhoPHis-R | 5’-GAGCATATGCGTATCCTTCT-3’  5’-GCCCTCGAGGCCCTCGTTGCGCGGA-3’ | This study  This study |
| PhoPN-F  PhoPN-R | 5’- TGTCTAGACTACGCGGCGATAA -3’  5’- GCAAGCTTCCAGGGTCGGCTT -3’ | This study  This study |
| PhoQN-F  PhoQN-R | 5’- ACCTCACCGAGCACATCTAC-3’  5’- CTGCCCGGCACGTCGAAGC-3’ | This study  This study |
| PhoQC-F  PhoQC-R | 5’- TGTCTAGATGCTGGAAAACG -3’  5’- AGAAGCTTAAGATCGAAACA -3’ | This study  This study |
| PhoP-F  PhoP-R  PhoQ-F  PhoQ-R | 5’- TGTCTAGATCAACCCGTACCC - 3’  5’- CCGGTACCGTCGGAAGAACCA -3’  5’- GGAAGCTTGAAGCCGATCGAGA-3’  5’- CAGGTACCAGCACTACCGATT-3’ | This study  This study  This study  This study |
| PhoPQ-F  PhoPQ-R  SodA1Q99-F  SodA1Q99-R  SodA2Q112-F  SodA2Q112-R  SodBQ95-F  SodBQ95-R  SodC1Q-F  SodC1Q-R  KatA1Q-F  KatA1Q-R  KatA2Q90-F  KatA2Q90-R  KatEQ95-F  KatEQ95-R  KatMnQ-F  KatMnQ-R  AhpCQ93-F  AhpCQ93-R  MacBQ-F  MacBQ-R  SmeZQ93-F  SmeZQ93-R  SmeVQ-F  SmeVQ-R  FadAQ-F  FadAQ-R  16S DNA-F  16S DNA-R | 5’-ACTCTAGATCAACCCGTACC -3’  5’-TCGGTACCAGCACTACCGATT -3’  5’- GATCCTCGCACTGGATGTCT-3’  5’- GCCCAGTTGATGATGTTGAA-3’  5’- ATCCATCACACCAAGCATCA-3’  5’- GCGACTTGAGCTTCTTCACC-3’  5’- GCACGCCTACTACACCGACT-3’  5’- GTAGCTGGCTCTCAGCGAAT-3’  5’- ACCGAACCTACTCCGCC-3’  5’- GCAGCGCCACCGGGATTGAAG-3’  5’- TCCATTCCCGACCCGACCACC-3’  5’- GCTTGCGCGTTTCCGCATCC-3’  5’- GCGTTCGGTACCTTCACTGT-3’  5’- GCGTTCGGTACCTTCACTGT-3’  5’- GGTGGCAGACAACCAGAACT-3’  5’- GGTCGAAGTGGGTGATCTTC-3’  5’- TCCAACCCGCAGCTCGCCAG-3’  5’- GCCCCAGCAGCCGCATATCCTC-3’  5’- CAGTGGTCCGTCCTGATCTT-3’  5’- CTTGAACTCGGCGTAATGGT-3’  5’- GCGAGATCATCGCCGACC-3’  5’- ACCACCGAGACCACCGAG-3’  5’- GATCCAGAAGGTGGTGCAGA-3’  5’- ATCAGCGTGTAGCGCCAGT-3’  5-GCGTGACAGCGAACTGCC-3’  5’-TCATCGATCAGCAGCGCC-3’  5’- GGAACACGTCTGCCTGCT-3’  5’-CTGCTGGATCGGCTTGTC-3’  5’- GACCTTGCGCGATTGAATG -3’  5’- CGGATCGTCGCCTTGGT -3’ | This study  This study  This study  This study  This study  This study  This study  This study  This study  This study  This study  This study  This study  This study  This study  This study  This study  This study  This study  This study  This study  This study  This study  This study  This study  This study  This study  This study  Chen et al., 2011  Chen et al., 2011 |

**References**

1. Hu, R.M., Huang, K.J., Wu, L.T., Hsiao, Y.J., Yang, T.C., 2008. Induction of L1 and L2 β-lactamases of *Stenotrophomonas maltophilia*. Antimicrob. Agents Chemother. 52, 1198-1200.
2. Simon, R., O'Connell, M., Labes, M., Puhler, A., 1986. Plasmid vector for the genetic analysis and manipulation of *Rhizobia* and other Gram-negative bacteria. Methods Enzymol. 118, 640-659.
3. Hoang, T.T., Karkhoff-Schweizer, R.R., Kutchma, A.J., Schweizer, H.P., 1998. A broad-host-range Flp-FRT recombination system for site-specific excision of chromosomally-located DNA sequences: application for isolation of unmarked *Pseudomonas aeruginosa* mutants. Gene. 212, 77-86.
4. Keen, N.T., Tamaki, S., Kobaysahi, D., Trollinger, D., 1998. Improved broad host-range plasmids for DNA cloning in gram-negative bacteria. Gene, 70, 191-197.
5. Chen, C.H., Huang, C.C., Chung, T.C., Hu, R,M,, Huang, Y.W., Yang, T.C. 2011. Contribution of resistance-nodulation-division efflux pump operon *smeU1-V-W-U2-X* to multidrug resistance of *Stenotrophomonas maltophilia*. Antimicrob. Agents Chemother. 55, 5826-5833.
