# Supplementary material for: PhoPQ two-component regulatory system plays a global regulatory role in antibiotic susceptibility, physiology, stress adaptation, and virulence in Stenotrophomonas maltophilia
Source: BMC Microbiol. 2020 Oct 14;20:312. doi: 10.1186/s12866-020-01989-z (PMC7559202; doi:10.1186/s12866-020-01989-z)
Supplement: Supplementary file 2 — Additional file 2: Figure. S1. SDS-PAGE and Western blotting of total bacterial proteins of S. maltophilia KJ, its derived mutants, and complementary strains. Overnight culture of bacterial cells was inoculated into fresh LB with an initial OD450nm of 0.15. Cells were grown aerobically for 5 h. Whole bacterial cell lysates were denatured and separated by 14% SDS-PAGE. (A) The gel was stained with 0.1% Coomassie Blue R250 in 10% acetic acid, 50% methanol, and 40% H2O for 30 min, and then destained with 10% acetic acid, 50% methanol, and 40% H2O until the background was clear. (B) The proteins in the gel were transferred onto polyvinylidene difluoride (PVDF) membranes. The blot was reacted with anti-PhoP antibodies and secondary mouse anti-rabbit IgG immunoglobulin conjugated to horseradish peroxidase (Sigma Chemical Co., St. Louis, MO). M, protein markers; lane 1, E. coli (pETPhoP); lane 2, KJ; lane 3, KJΔPhoP; lane 4, KJΔPhoP (pPhoP); lane 5, KJΔPhoP (pPhoPQ); lane 6, KJΔPhoPQ; lane 7, KJΔPhoPQ (pPhoP); lane 8, KJΔPhoPQ (pPhoQ); lane 9, KJΔPhoPQ (pPhoPQ). The cropped image presented in Fig. 2b is marked by a black rectangle. [file 12866_2020_1989_MOESM2_ESM.docx]

**M 1 2 3 4 5 6 7 8 9**

**(A)**


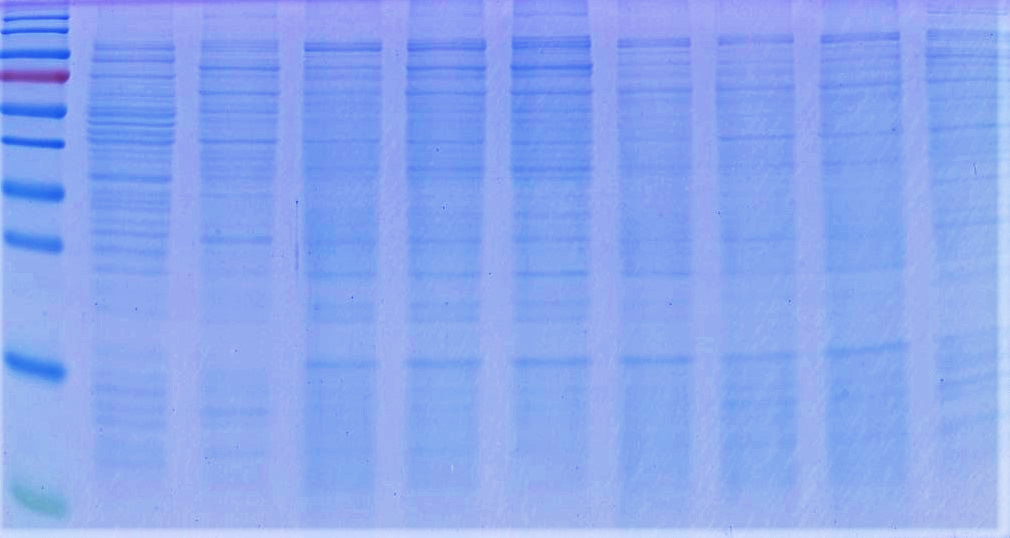


**25 KDa**

**1 2 3 4 5 6 7 8 9**

**(B)**





**PhoP**

**Fig. S1. SDS-PAGE and Western blotting of total bacterial proteins of *S. maltophilia* KJ, its derived mutants, and complementary strains.** Overnight culture of bacterial cells was inoculated into fresh LB with an initial OD_450nm_ of 0.15. Cells were grown aerobically for 5 hours. Whole bacterial cell lysates were denatured and separated by 14% SDS-PAGE. (A) The gel was stained with 0.1% Coomassie Blue R250 in 10% acetic acid, 50% methanol, and 40% H_2_O for 30 min, and then destained with 10% acetic acid, 50% methanol, and 40% H_2_O until the background was clear. (B) The proteins in the gel were transferred onto polyvinylidene difluoride (PVDF) membranes. The blot was reacted with anti-PhoP antibodies and secondary mouse anti-rabbit IgG immunoglobulin conjugated to horseradish peroxidase (Sigma Chemical Co., St. Louis, MO). M, protein markers; lane 1, *E. coli*(pETPhoP); lane 2, KJ; lane 3, KJΔPhoP; lane 4, KJΔPhoP(pPhoP); lane 5, KJΔPhoP(pPhoPQ); lane 6, KJΔPhoPQ; lane 7, KJΔPhoPQ(pPhoP); lane 8, KJΔPhoPQ(pPhoQ); lane 9, KJΔPhoPQ(pPhoPQ). The cropped image presented in Fig. 2B is marked by a black rectangle.
