# Supplementary material for: PhoPQ two-component regulatory system plays a global regulatory role in antibiotic susceptibility, physiology, stress adaptation, and virulence in Stenotrophomonas maltophilia
Source: BMC Microbiol. 2020 Oct 14;20:312. doi: 10.1186/s12866-020-01989-z (PMC7559202; doi:10.1186/s12866-020-01989-z)
Supplement: Supplementary file 3 — Additional file 3: Figure S2. The swimming motility of wild-type KJ, phoP mutant (KJΔPhoP), phoPQ mutant (KJΔPhoPQ), and complementation strain (KJΔPhoPQ (pPhoPQ)). The logarithmic-phase bacterial culture was adjusted to OD450nm of 1.0. Five-microliter suspension was inoculated into the swimming agar (1% tryptone, 0.5% NaCl, and 0.15% agar) and the swimming zones were recorded after 48-h incubation at 37 °C. [file 12866_2020_1989_MOESM3_ESM.docx]

**KJΔPhoPQ**

**KJ**

**KJΔPhoPQ(pPhoPQ)**

**KJ**

**KJΔPhoP**

**Fig. S2. The swimming motility of wild-type KJ, *phoP* mutant (KJΔPhoP), *phoPQ* mutant (KJΔPhoPQ), and complementation strain (KJΔPhoPQ(pPhoPQ)).** The logarithmic-phase bacterial culture was adjusted to OD_450nm_ of 1.0. Five-microliter suspension was inoculated into the swimming agar (1% tryptone, 0.5% NaCl, and 0.15% agar) and the swimming zones were recorded after 48-h incubation at 37°C.
