# Supplementary material for: PhoPQ two-component regulatory system plays a global regulatory role in antibiotic susceptibility, physiology, stress adaptation, and virulence in Stenotrophomonas maltophilia
Source: BMC Microbiol. 2020 Oct 14;20:312. doi: 10.1186/s12866-020-01989-z (PMC7559202; doi:10.1186/s12866-020-01989-z)
Supplement: Supplementary file 4 — Additional file 4: Figure S3. The secreted protease activity of wild-type KJ, phoP mutant (KJΔPhoP), phoPQ mutant (KJΔPhoPQ), and complementation strain (KJΔPhoPQ (pPhoPQ)). The logarithmic-phase of bacterial culture was adjusted to OD450nm of 1.0 and 40 μl bacterial aliquot were inoculated into the skim milk agar plate. The proteolytic activity of bacteria was assessed by measuring the transparent zones around the bacteria after incubation for 72 h at 37 °C. [file 12866_2020_1989_MOESM4_ESM.docx]

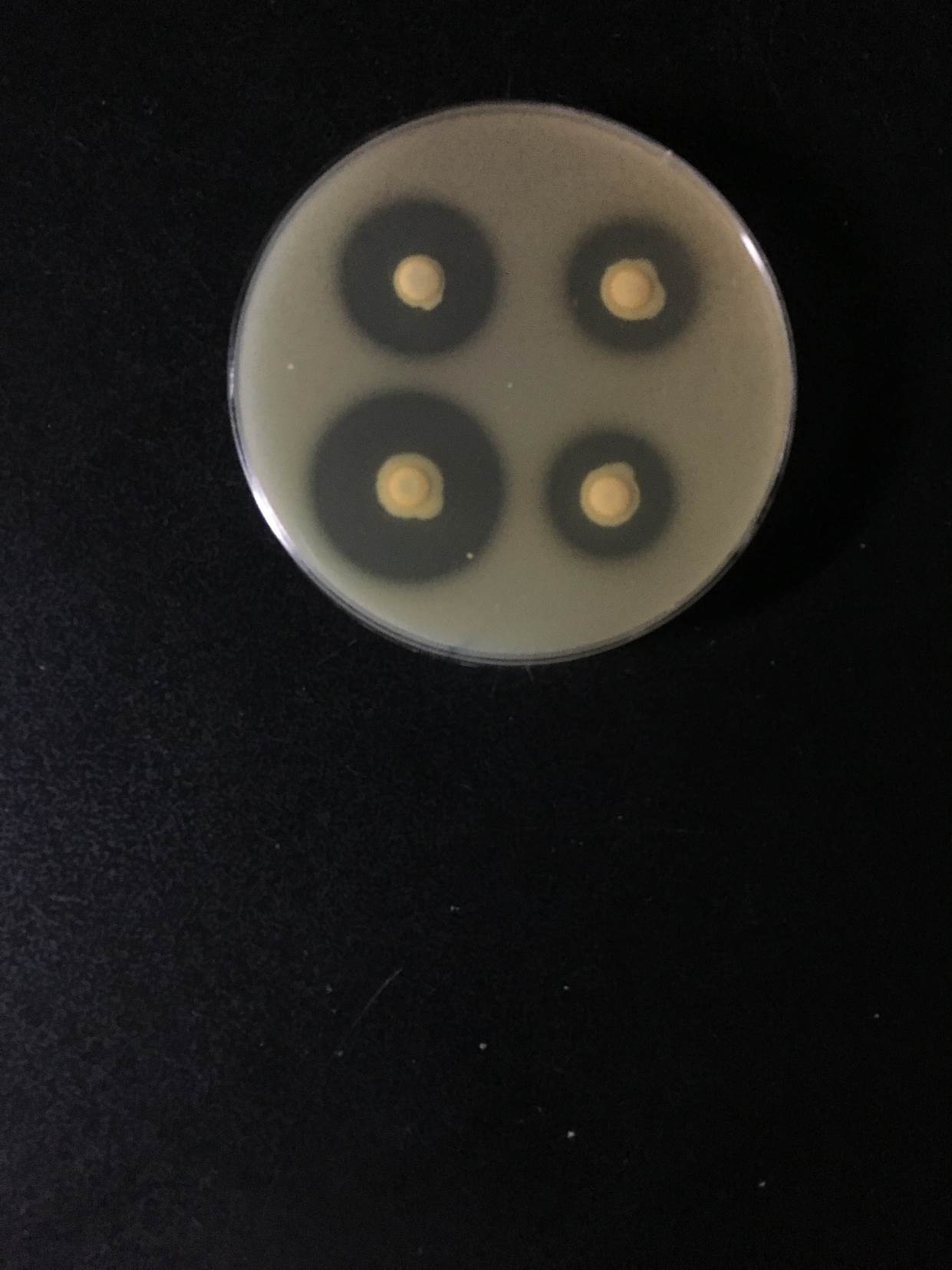


**KJ**

**KJΔPhoP**

**KJΔPhoPQ**

**KJΔPhoPQ**

**(pPhoPQ)**

**Fig. S3. The secreted protease activity of wild-type KJ, *phoP* mutant (KJΔPhoP), *phoPQ* mutant (KJΔPhoPQ), and complementation strain (KJΔPhoPQ(pPhoPQ)).** The logarithmic-phase of bacterial culture was adjusted to OD_450nm_ of 1.0 and 40 μl bacterial aliquot were inoculated into the skim milk agar plate. The proteolytic activity of bacteria was assessed by measuring the transparent zones around the bacteria after incubation for 72 h at 37°C.
