# Supplementary material for: PhoPQ two-component regulatory system plays a global regulatory role in antibiotic susceptibility, physiology, stress adaptation, and virulence in Stenotrophomonas maltophilia
Source: BMC Microbiol. 2020 Oct 14;20:312. doi: 10.1186/s12866-020-01989-z (PMC7559202; doi:10.1186/s12866-020-01989-z)
Supplement: Supplementary file 5 — Additional file 5: Figure S4. The expression of oxidative stress alleviation-associated genes of S. maltophilia KJ in response to MD challenge. Overnight culture of KJ cells was inoculated into fresh LB broth without or with MD (16 μg/ml) at an initial OD450nm of 0.15. Cells were grown aerobically for 5 h before measuring the indicated transcripts using qRT-PCR. All values were normalized to the transcript of MD-non-treated KJ cells. Bars represent the average values from three independent experiments. Error bars represent the standard error of the mean. *, P < 0.001, significance calculated by Student’s t test. [file 12866_2020_1989_MOESM5_ESM.docx]

**200**

*****

**150**

* *

**100**

**KJ**

**KJ + MD**

**KJ**

**KJ + MD**

**KJΔPhoPQ + MD**

*****

**50**

**Relative expression level (Fold)**

*

*****

*****

**6**

**5**

**4**

**3**

**2**

**1**

**0**

*****

*****

*

*

* *

* *

***smeV***

***smeZ***

***ahpC***

***macBsm***

***fadA***

***katMn***

***katE***

***katA2***

***katA1***

***sodB***

***sodC***

***sodA2***

***sodA1***

**Fig. S4. The expression of oxidative stress alleviation-associated genes of *S. maltophilia* KJ in response to MD challenge.** Overnight culture of KJ cells was inoculated into fresh LB broth without or with MD (16 μg/ml) at an initial OD_450nm_ of 0.15. Cells were grown aerobically for 5 h before measuring the indicated transcripts using qRT-PCR. All values were normalized to the transcript of MD-non-treated KJ cells. Bars represent the average values from three independent experiments. Error bars represent the standard error of the mean. *, *P* < 0.001, significance calculated by Student’s t test.
